# The Relationship between Odour Annoyance Scores and Modelled Ambient Air Pollution in Sarnia, “Chemical Valley”, Ontario

**DOI:** 10.3390/ijerph6102655

**Published:** 2009-10-16

**Authors:** Dominic Odwa Atari, Isaac N. Luginaah, Karen Fung

**Affiliations:** 1 Department of Geography, University of Western Ontario, London, Ontario, N6G 5C2, Canada; E-Mail: iluginaa@uwo.ca; 2 Department of Mathematics and Statistics, University of Windsor, Windsor, Ontario, N9B 3P4, Canada; E-Mail: kfung@uwindsor.ca

**Keywords:** land use regression, odour annoyance, pollution, nitrogen dioxide, sulphur dioxide, Sarnia, Ontario

## Abstract

This study aimed at establishing the relationship between annoyance scores and modelled air pollution in “Chemical Valley”, Sarnia, Ontario (Canada). Annoyance scores were taken from a community health survey (N = 774); and respondents’ exposure to nitrogen dioxide (NO_2_) and sulphur dioxide (SO_2_) were estimated using land use regression (LUR) models. The associations were examined by univariate analysis while multivariate logistic regression was used to examine the determinants of odour annoyance. The results showed that odour annoyance was significantly correlated to modelled pollutants at the individual (NO_2_, r = 0.15; SO_2_, r = 0.13) and census tract (NO_2_, r = 0.56; SO_2_, r = 0.67) levels. The exposure-response relationships show that residents of Sarnia react to very low pollution concentrations levels even if they are within the Ontario ambient air quality criteria. The study found that exposure to high NO_2_ and SO_2_ concentrations, gender, and perception of health effects were significant determinants of individual odour annoyance reporting. The observed association between odour annoyance and modelled ambient pollution suggest that individual and census tract level annoyance scores may serve as proxies for air quality in exposed communities because they capture the within area spatial variability of pollution. However, questionnaire-based odour annoyance scores need to be validated longitudinally and across different scales if they are to be adopted for use at the national level.

## 1. Introduction

The assessment of exposure to traffic and industry related pollution continues to be a challenge. Most epidemiological studies that assess health effects of pollution use fixed monitoring sites and different modelling techniques (e.g., kriging, dispersion), but adequate information and data are not always available [1]. For example, kriging has been used both at the national and regional scale [2], but it has been criticised for its inability to capture air pollution at very short distances [3]. Other estimation techniques such as microenvironment monitoring have been hampered by high costs related to data collection especially when dealing with a large cohort [4,5]. Furthermore, the use of traditional dispersion models have been restricted because of their expensive data demands and lack of precision in the requisite meteorological or emissions data required for making accurate predictions [6,7].

Recently, land use regression (LUR) modelling has been proposed as an alternative approach to address some of the limitations by assessing the intra-urban spatial variability of ambient air pollutants in urban and industrial settings. LUR modelling captures localized variations in ambient air pollution more effectively and economically than some of the conventional approaches discussed above [2]. In addition, LUR modelling predicts ambient air pollution concentrations at given sites based on the surrounding land use, traffic, population and dwelling counts, and physical characteristics such as elevation [6]. Several researchers [2,6,8] have provided critical reviews of LUR studies and emphasized its potential role in estimating exposure to outdoor air pollution.

Annoyance caused by air pollution has been suggested as an indicator for ambient air pollution exposure [9,10]. This exposure estimation technique incorporates broader scopes and domains such as quality of life and community values [10,11]. Invariably, there are contrasting reports showing the relationship between annoyance scores and modelled ambient exposures. For example, while examining the predictors of perceived annoyance from air pollution in six European cities (Athens, Basel, Milan, Oxford, Prague, and Helsinki), Rotko *et al.* [12] found no association between outdoor nitrogen dioxide (NO_2_) pollution levels and annoyance scores at individual levels. In Sweden, Forsberg *et al.* [13] reported no significant association between sulphur dioxide (SO_2_) and annoyance scores. When comparing self-reported traffic intensity to modelled air pollution from traffic in three birth cohorts from three countries: the Netherlands; Germany; and Sweden, Heinrich *et al.* [14] found weak association between the subjective self-reported assessments of exposure and NO_2_ modelled estimates. In addition, while examining the relationship between publicly available air quality data and public perception of air quality in London, UK, Williams and Bird [11] reported that perception of pollution exposure was not consistent with air quality data for urban and suburban areas although there were some trends with women and older people perceiving higher levels of air pollution than their female and younger counter parts.

On the other hand, while examining whether a questionnaire-based indicator (annoyance) of ambient air pollution can be a useful proxy for assessing the within-area variability of air quality in Switzerland, Oglesby *et al.* [15] reported a strong association between annoyance and modelled NO_2_ concentration at home, but also found that smoking, workplace dust exposure, and respiratory symptoms were significant predictors of individual annoyance score. While Forsberg *et al.* [13] reported a lack of association between annoyance and SO_2_, they did report a high correlation between NO_2_ concentration and annoyance related to air pollution and traffic exhaust fumes. Furthermore, Jacquemin *et al.* [16] reported an association between self-reported annoyances caused by ambient air pollution and outdoor NO_2_ concentration levels in 20 cities from 10 European countries. They concluded that annoyance scores could be a useful measure of perceived outdoor air quality. Smith *et al.* [17] found that the degree of concern voiced about foul air was closely related to the level of ambient air pollution experienced by their study subjects in Nashville, Tennessee. Similarly, Modig and Forsberg [18] reported a significant increase of people self-assessed annoyance with rising levels of modelled NO_2_ concentrations in three Swedish cities (Umea, Uppsala, and Gothenburg). In Oslo, Norway, Piro *et al.* [9] found that annoyance to air pollution problems are strongly associated with increased levels of modelled air pollution concentrations

This study extends the emerging literature on the relationship between odour annoyance and air pollution in Sarnia “Chemical Valley”, Ontario (Canada), a sentinel high exposure environment; with the following specific objectives: a) to determine the correlations between odour annoyance score and modelled NO_2_ and SO_2_ at individual and census tract levels; b) to examine the individual determinants of odour annoyance caused by industrial pollution; and c) to establish exposure–response relationship between NO_2_ and SO_2_ exposure and odour annoyance.

## 2. Theoretical Context

This study is informed by risk perception and odour annoyance literature [19–21]. In general, health risk perception plays an important role on how individuals and the public respond to environmental exposures. While examining people's perceptions of problems and social cohesion in neighbourhoods in Quebec City, Quebec, Pampalon *et al.* [22] found that perceptions of place appear to be significant predictors of health and well-being. Likewise, in Glasgow, Ellaway *et al.* [23] reported that self-rated health is associated with perceived neighbourhood problems and cohesion. In Hamilton, Ontario, Elliott *et al.* [24] found that the relationships between environmental exposure and health outcomes are mediated by risk perception of exposure (e.g., air pollution); and that they cannot be divorced from the wider community context in which they occur. However, there are observed discrepancies between lay persons’ perception of environmental and technological risks and those of the scientific and policy experts on the difference between “reality” and perception [19]. These differences have raised concern and even perplexity among those responsible for the management of environment risk.

Scientists assume they have more objective understanding of risk due to their rigorous experimental studies, epidemiological surveys, and probabilistic risk analysis; but the lay persons’ understanding of risk is based on misperceptions or misunderstandings of the objective (real) risk [19]. However, some studies have reported that lay people are not ignorant of what is real risk but (when compared to experts) lay persons employ a broader and richer kind of rationality influenced by complex social, political, and cultural processes to assess exposure to environmental risk [19]. Lay persons take into consideration qualitative issues such as future generation and their personal lives, and bring in their beliefs and values to judge the “reality” of environmental risk [21]. Hence, lay persons’ perception of air pollution might be real because they depend in part on the pollution concentrations levels they are exposed to. Consequently, self-reported annoyance may correlate with monitored or modelled exposure. Thus, public concerns could not be blamed on ignorance or irrationality but rather to the sensitivity to technical, social, and psychosocial qualities of hazards that are not well-modelled in technical risk assessment [21].

Odours from industrial sources, such as the petrochemical plants in Sarnia, have been shown to considerably impact general health and well-being by affecting both the physiological and psychosocial status of people [20,25]. Such impacts are reinforced when air pollution odours are absorbed by building materials and then released slowly overtime [26,27]. Shusterman *et al.* [20] argued that odours appear to contribute to lay people’s judgment of environmental air quality, and provide important diagnostic information in appraising the potential threats to health and well-being. As a result, perceptual ideas about toxicity of environmental pollution seem to suggest that “if environments smell bad, they’re probably damaging to health” [28, p. 412] or at the very least, they might cause annoyance – feelings of displeasure associated with agents or conditions believed to have adverse effects on an individual or groups of individuals. Neutra *et al.* [29] identified odour annoyance as a powerful effect modifier in several studies of symptom rates around hazardous waste sites which can be extended to a highly exposed environment such as Sarnia because of the numerous petrochemical industries in the region. Annoyance responses are modified by personal factors and community level factors, such as age, gender and perceived health status [9,26,30], attitudes toward the exposure source, and individual sociodemographic characteristics [13,26]. For example, Forsberg *et al.* found that the frequency of reporting annoyance was high among women and people with respiratory illnesses like asthma. These discussions above provide a background for interpreting the relationship between modelled pollution and annoyance, and the determinants of annoyance within the context of a highly exposed environment.

## 3. Methods

### 3.1. Study Area

**Figure 1.**
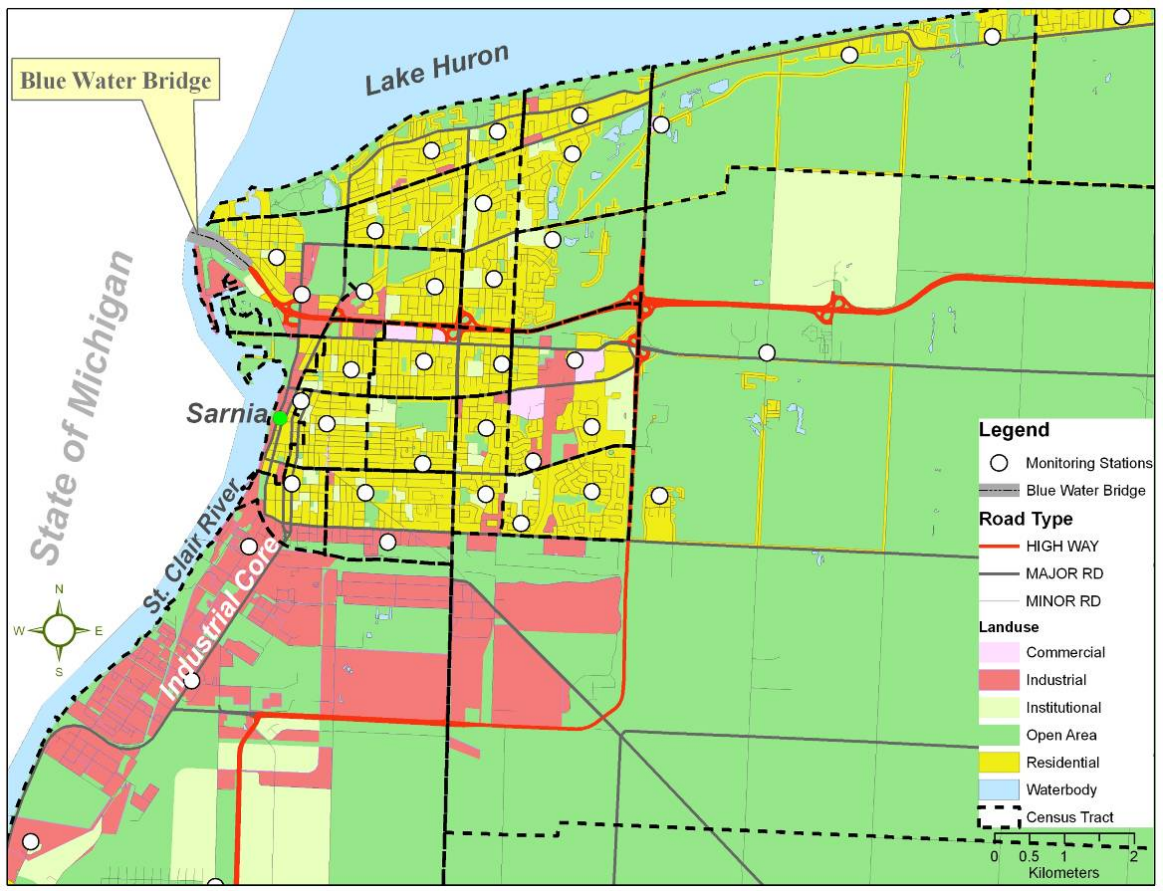
Study area.

Sarnia is located in Southwestern Ontario, Canada, and has an approximate land area of 165 km^2^ and a population of 71,419 [31]. The City of Sarnia and its surrounding communities (Figure 1) are called ‘Chemical Valley’ because they are the center of more than 40% of Canada’s chemical industries including, for example, Suncor, Bayer, Dow Canada, NOVA, and ESSO. One of the largest and well known landfill sites in Canada, Safety-Kleen Inc, is also located in Chemical Valley.

### 3.2. Study Design

As part of a larger community health study aimed at examining the determinants of health in Sarnia, a multi-method approach including qualitative and quantitative approaches were used. Detailed descriptions of these approaches are found elsewhere [32–34]. In brief, the qualitative study examined Sarnia residents’ daily lived experiences, perceptions of and responses to living within a government labelled “Area of Concern” (AOC). Findings from the qualitative study were used to develop a community health survey instrument. In total, 804 residents of Sarnia were randomly surveyed using a computer assisted telephone interview system in October 2005. The survey contained a range of questions that captured residents’ attitudes toward the local area, general health status, chronic conditions, perceptions of air pollution, personal health behaviour, occupational exposure, coping, and socio-demographic data. The survey was completed by residents aged 18 years and older whose birthdays were closest to the day of the survey [35]. While the survey was administered, several ambient air pollutants including NO_2_ and SO_2_ were monitored at 39 locations across the city of Sarnia for two weeks. LUR was utilized to model the intra-urban spatial variability of measured ambient NO_2_ and SO_2_ concentrations [33]. Figure 1 shows the study area with the distribution of land use, road types, and locations of monitoring stations used for the modelling. Approval for this study was granted by the University of Western Ontario ethics committee.

### 3.3. Study Variables

#### 3.3.1. Independent variables

The developed LUR model equations [33] were used to estimate individual’s exposures to modelled NO_2_ and SO_2_ pollutants based on their 6-digit postal codes. As covariates, indoor exposures were measured by asking respondents the number of indoor appliances including air humidifiers, filters and conditioners they have in their dwellings. Residents were also asked whether they were exposed to dust or fumes at home or work.

The influence of five health variables including self-assessed health status, chronic conditions, emotional distress, cardinal, and general health symptoms were examined. Self-reported health status [36] was assessed by asking respondents “In general, compared to other people your age, would you say your health is: ‘excellent’, ‘very good’, ‘good’, ‘fair’ or ‘poor’?” The variable was categorised into ‘excellent’, ‘very good’, and ‘good’ verses ‘fair’ and ‘poor’ health status. Emotional distress was assessed by using the 20-item version of the General Health Questionnaire (GHQ), a validated measure of emotional well-being [37]. For each of the GHQ item, respondents indicated if they felt a certain way (for example: unable to concentrate, reasonably unhappy, feeling nervous and tense) in the past two weeks. For analysis, all the 20 items in the GHQ were summed and dichotomized into individuals with emotional distress (GHQ ≥ 4) and those with no emotional problems (GHQ < 4) [36,37]. In addition, respondents were asked about the presence or absence of 13 physician diagnosed chronic conditions such as diabetes, cancer, asthma, and arthritis [38]. The responses to the chronic conditions were dichotomized into individuals with one or none (as a reference category) verses those with two or more chronic conditions. Cardinal symptoms were health symptoms including coughs, wheezing/breathing problems, nausea, sinus congestion, colds, skin rashes, eye, nose, or throat irritations, earaches, and nosebleeds which are more likely caused by the irritant properties of pollution. On the other hand, general health symptoms were health indicators which were more likely to result from stress-mediated mechanisms related to odour annoyance and they include symptoms such as headaches, sleep problems, dizzy spells, stomach aches, diarrhea, loss of appetite, and chest pains. Due to the random occurrence of health symptoms (e.g., nausea and nasal congestion) in the general population [26,39], we dichotomized these health symptoms (e.g., two or less vs three or more symptoms) for all respondents.

#### 3.3.2. Dependent variable: degree of annoyance

Study respondents self-assessed their degree of annoyance due to air pollution odours on an 11-point annoyance indication (0: no disturbance at all, 10: intolerable disturbance) through the following question: “How much are you annoyed by odours from the chemical plants at your actual home, if you keep the windows open?” Similar scale has been validated in other European studies [15].

### 3.4. Statistical Analysis

Both univariate and multivariate approaches were used to examine the relationship between annoyance score and the LUR modelled exposures at the individual and census tract levels. The individual level measurements are respondent’s records of annoyance score or estimated exposure while census tract level measurements are mean values of all individual annoyance scores or estimated exposure that fall within each census tract—subdivision of a county. Annoyance scores at the individual levels were related to gender, age, and estimated pollution quartiles (low: 1^st^ quartile, moderate: 2^nd^ and 3^rd^ quartiles, and high: 4^th^ quartile). In addition, the continuous individual and census tract mean annoyance scores were correlated and regressed against the individual and census tract mean pollution estimates. In total, there were 20 valid census tracts (out of 24) that had individuals whose mean annoyance scores and pollution estimates were determined. From the total sample, 30 respondents had missing annoyance records, and they were dropped from the analysis (leaving a total sample of 774).

**Figure 2.**
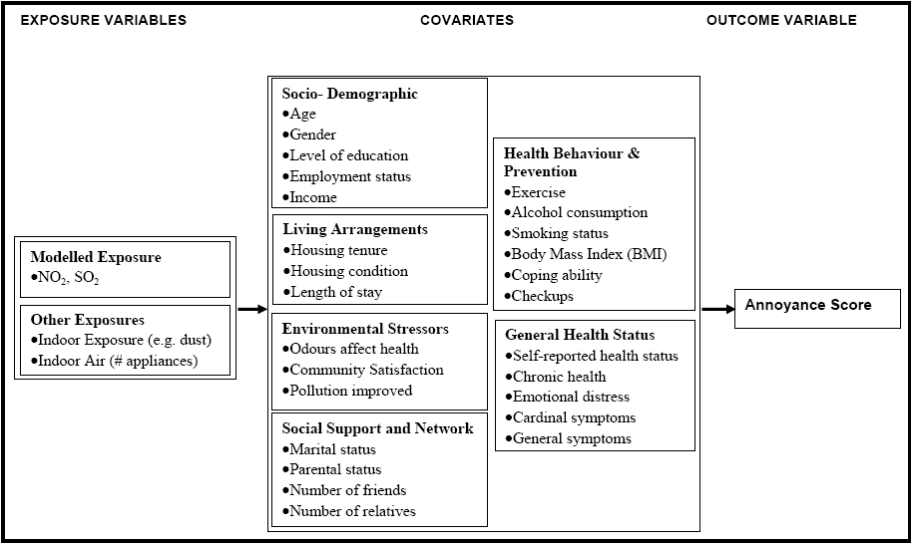
Analytical framework.

Multivariate logistic regression analysis was used to examine the determinants of annoyance score at the individual level. The theoretical contexts explained above inform the analytical model used in this study. Essentially, the model (Figure 2) is composed of three main components: exposure variables—reported, measured and modelled exposure related variables [13,15]; covariates—those variables which influence odour annoyance reporting, for example, general health variables, sociodemographic variables [9,26,30]; and odour annoyance as an outcome variables [13,15]. Mediator variables (covariates) including: sociodemographic variables (sex, age, educational level, employment and income); living arrangements (housing tenure and condition, years in community), environmental stressors (perception of odours and community satisfaction) were examined as potential modifiers to odour annoyance reporting. Social support and social network variables that were included in our assessments were marital and parental statuses, number of friends and number of relatives. Personal health behaviour and prevention (exercise, drinking alcohol, smoking status, body mass index (BMI), coping skills, and medical checkups) and general health status were also assessed (Figure 2). The annoyance scale was dichotomised into “high annoyance” (scores ≥ 8) and “low annoyance” (scores < 8) ratings [15]. A hierarchical model was built by entering each block of explanatory variables systematically as shown in the analytical model: exposure variables and covariates (Figure 2). The variables which made significant contributions to the model at each stage were retained. Variables were judged to contribute to the model if the significance level of the Wald inclusion test statistic was 0.10 or lower; or the significance level was greater than 0.10 but a contribution to the model was indicated via a partial correlation greater than zero and/or an improvement in the percentage of respondents correctly classified. Models were run using a stepwise backward elimination algorithm within each block. Due to their *a priori* importance, age and gender were forced into every model regardless of their contribution. First order interaction terms were entered into the model using forward-stepwise selection.

To examine the exposure—response relationships between modelled NO_2_ and SO_2_ exposures and odour annoyance, ordinal logit models were used because the dependent variable—odour annoyance—was categorical [40]. The models were used to generate parameter estimates for two thresholds: little annoyed (annoyance score = 1–7) and highly annoyed (annoyance score >= 8) and the individual modelled pollution concentrations as the location parameter [41]. Similar to Amundsen *et al.* [41], we used Equation (1) to obtain the estimated exposure – response relationships from the estimated parameters. The equation indicates the probability of obtaining odour annoyance response higher or equal to *j*:

(1) $$P(\text{Y}\geq j|{\text{X}}_{i}={\text{x}}_{i})=1-(({\text{e}}^{\tau j-\beta \text{x}i})/(1+{\text{e}}^{\tau j-\beta \text{x}i}))\;J\;\in [1,\ldots ,J-1]$$

where τ*_j_* indicates the *j*th estimated threshold, and β is the estimated parameter for the exposure value. There are *J* odour annoyance categories. X*_i_* is a vector of exposure and modifying variables for an individual *i*.

## 4. Results

The mean LUR modelled pollution concentrations for individuals (N = 774) was 13.82 ± 1.64 ppb for NO_2_ and 3.17 ± 1.53 ppb for SO_2_. The 24 hour ambient air quality criteria (AAQC) developed by the Ontario Ministry of Environment [42] for NO_2_ and SO_2_ concentrations was “100 ppb”. The correlation coefficients between modelled NO_2_ and SO_2_ were 0.49 and 0.65 at individual and census tract levels, respectively. Overall, 34% of respondents reported no annoyance at all; 50% reported little annoyance (1–7); and 16% reported high annoyance (≥8). The overall mean annoyance was 3.22 with a median of 2. Table 1 shows the distribution of annoyance scores by modelled pollutants and gender.

The results show that annoyance scores increase with increasing NO_2_ and SO_2_ pollution concentrations. Female respondents reported comparatively higher mean annoyance scores than their male counter parts. The results also showed a wide range of perception at a given level of ambient air pollution based on age group (Table 2). When the mean annoyance scores were compared, age groups showed a trend with older people less annoyed than the younger ones with the exception for the 18–24 group. In general, the mean annoyance score corresponded to the gradient of the modelled pollutants despite age differences.

The Pearson correlation coefficients between odour annoyance and modelled pollutants were all positive and significant but low for NO_2_ (r = 0.15) and SO_2_ (r = 0.13) at the individual level compared to the high coefficients of 0.56 and 0.67 at the census tract level for NO_2_ and SO_2_, respectively (Table 3). The univariate linear regression analyses indicate that modelled NO_2_ and SO_2_ concentrations each explained only 2% of the annoyance scores variance at the individual level but 32 and 44% of the variances at the census tract level, respectively (Table 4).

**Figure 3.**
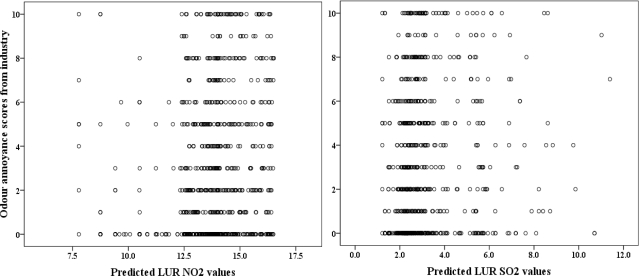
Relationship between individual annoyance scores and modelled NO_2_ and SO_2_ concentrations.

**Figure 4.**
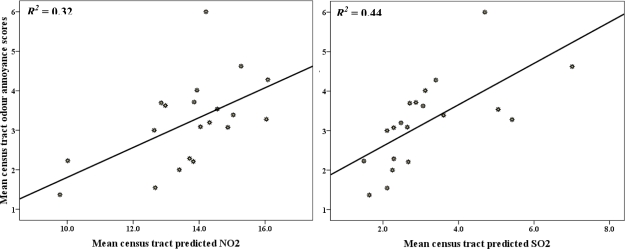
Relationship between mean census tract annoyance score and predicted NO_2_, and SO_2_.

Figure 3 shows the scatterplot between modelled pollutant concentrations and annoyance scores observed at an individual level; while Figure 4 shows the distribution and lines of best fit between mean census tract odour annoyance scores and modelled NO_2_ and SO_2_ concentrations. The results show that annoyance score is better predicted at the census tract than the individual level.

In the multivariate logistic regression analysis, each possible determinant was evaluated one at a time, and a dichotomized odour annoyance rating (annoyance score ≥ 8 versus < 8) of industrial odour disturbances was used as an outcome variable. We examined the influence of modelled NO_2_ and SO_2_ on annoyance reporting separately. Table 5 shows the relationship between high annoyance and modelled NO_2_ concentrations and also together with other covariates. When age and gender were controlled (Model II, Table 5), the 3^rd^ and 4^th^ high NO_2_ quartiles retained significant influence on high odour annoyance reporting. Age did not show any significance influence on high annoyance reporting but age groups reflected annoyance score gradient with older people reporting less likelihood of high annoyance score. Gender was significant with females more likelihood to report high annoyance than their male counter parts (OR = 2.17, p-value < 0.001). In model III, the introduction of occupational exposure to dust made significant effect on odour annoyance score reporting with those more exposed at work more likely to report odour annoyance (OR = 1.57, p-value < 0.05). When sociodemographic variables, general health status (i.e. health status, cardinal symptoms), and perception of odours were controlled (Model IV), the odd ratios of reporting high odour annoyance increased for people exposed to high NO_2_ pollution quartiles. Residents exposed to the 3^rd^ and 4^th^ NO_2_ concentrations quartiles were more than 3 times more likely to report high annoyance than residents who are exposed to the 1^st^ and 2^nd^ pollution concentrations quartiles. In the final model (Model V, Table 5), high pollution concentration, gender, odour perception (that odours impact health and have not improved in the last 5 years), and the ability to cope with day-to-day demands showed significant influence on reporting of high odour annoyance. The multivariate modelling suggests that the relationship between odour annoyance and NO_2_ concentrations was influenced by gender, cardinal symptoms, and odour perception. The inabilities to cope with day-to-day demands also influence high odour annoyance reporting (OR = 2.06, p-value < 0.05). With the first order interaction, the final NO_2_-based model showed an improved model with acceptable goodness of fit (ρ^2^) of 0.21 [43]. The goodness of fit is defined as one minus the ratio of the maximum log likelihood values of the fitted and constant only-term (null) models. Calculated values for goodness of fit range from zero to one, and values between 0.2 and 0.4 represent a very good fit of the model [43].

The relationship between modelled SO_2_ concentrations and high odour annoyance scores remained significant even after we controlled for age and gender (Model II, Table 6). Controlling for occupational exposure to dust (Model III, Table 6) moderately increased the influence of SO_2_ concentrations on high odours annoyance reporting (OR = 1.57, p-value < 0.05). Upon the inclusion of sociodemographic variables, general health status (i.e. health status, cardinal symptoms), and perception of odours, the 3^rd^ and 4^th^ SO_2_ concentrations quartiles gained strength as predictors for high odour annoyance reporting (Model IV, Table 6). Residents exposed to high SO_2_ concentrations were more than 4 times more likely to report high odour annoyances. However, the influence of gender was weakened (odds ratio reduced from 2.06 in the initial model to 1.71 in the final model) but remained significant. Individuals who reported more than two cardinal symptoms were more than two times more likely to report high odour annoyance. Respondents who believed that odours would adversely impact their health were more than five times more likely to report high annoyance compared to individuals who were neutral or are in disbelieve that industrial odours will impact their health (OR = 5.33, p-value < 0.001).

In addition, residents who perceive that odours have not improved in the last five years were 80% more likely to report high annoyance than individuals who believed that odours related to the industrial pollution in their region have improved (OR = 1.80, p-value < 0.05). When the first order interaction effects were introduced into the model, the high SO_2_ concentrations quartiles maintained their influence on high odour annoyance (Model V, Table 6). The interaction effects showed that residents who are exposed to occupational dust and are reporting more than two cardinal symptoms are almost five times more likely to report high odour annoyance (OR = 4.79, p-value < 0.01). The final SO_2_-based model showed satisfactory goodness of fit (ρ2 = 0.21), Cox and Snell R2 (0.17), and Nagelkerke R2 (0.29) [43].

In general, NO_2_ and SO_2_ pollutions have significant influence in high odour annoyance reporting even in the context of other covariates. The overall contributory effects of the mediating variables suggest that these variables may be exacerbating the impact of pollution in self-reporting of high odour annoyance.

Table 7 shows the results from the ordinal logit model parameter estimations. These results are used to calculate the estimated exposure – response relationship in equation (1). For example, to calculate the proportion of respondents who are estimated to be annoyed by 10 ppb of NO_2_ concentration level, the estimated parameters values (Table 7) of the relevant threshold and location (exposure) parameter were inserted into the expression as follows:

$$P(\text{Y}\geq j|{\text{X}}_{i}=10)=1-(({\text{e}}^{4.399-10\;\times \;0.196})/(1+{\text{e}}^{4.399-10\;\times \;0.196}))\approx 0.08$$

**Figure 5.**
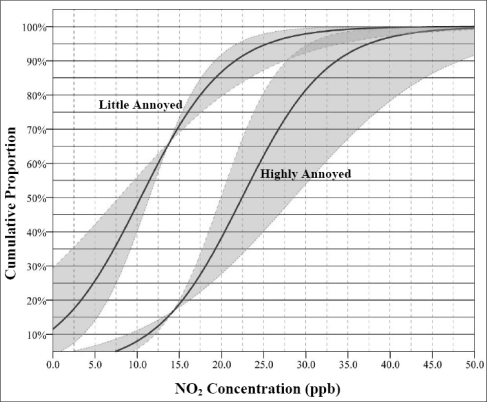
Cumulative exposure–response curve for NO_2_ and the proportion of respondents who express different degrees of odour annoyance.

**Figure 6.**
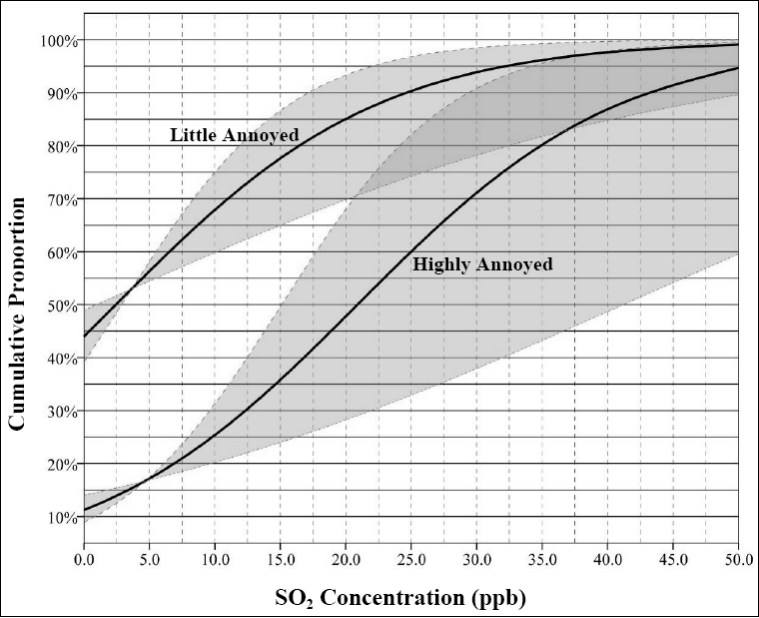
Cumulative exposure–response curve for SO_2_ and the proportion of respondents who express different degrees of odour annoyance.

The result shows that about 8% of the respondents are highly annoyed at 10 ppb NO_2_ exposure level. Figure 5 and 6 show the exposure – response relationship for NO_2_ and SO_2_ in the Sarnia respectively. The lower curve indicates the percentage of residents expected to be *highly annoyed* by given exposure levels from the industries. The upper curve is cumulative percentage of respondents who are at least a *little annoyed*. The gray bands indicate the 95% confidence intervals of the relationships between exposure and annoyance. These figures show quite large individual variation in reporting odour annoyance at given pollution levels (Figures 5 and 6). The figures indicate that, for example, at a 20 ppb NO_2_ concentration levels, about 87% of respondents are at least a little annoyed while 37% of them are highly annoyed by industrial odour in their home addresses (Figure 5).

With the same concentration of SO_2_ (20 ppb), at least 85% of respondents are a little annoyed and more than 47% are highly annoyed by odours in the region (Figure 6). The results show that many people are highly annoyed by SO_2_ (47%) compared to NO_2_ pollution (37%) at the same pollution concentration levels. At the same pollution levels, about 87 and 85% of Sarnia residents are at least a little annoyed to NO_2_ and SO_2_ pollution respectively. It should be noted that there are few or no concentrations for NO_2_ above 17 ppb and above 12 ppb for SO_2_ modelled concentrations. The range of 0–50 ppb was used to facilitate comparisons.

## 5. Discussion and Conclusions

This study examines the relationship between odour annoyance and modelled pollution, exposure– response associations, and the determinants of annoyance scores in the highly exposed environment of Sarnia. Although modelled NO_2_ and SO_2_ concentrations are below Ontario’s AAQC, a considerable proportion of residents surveyed were annoyed by the odours. The study found a strong association between annoyance scores and the different LUR modelled ambient air pollutants including NO_2_ and SO_2_. Consistent with other studies [15,16], people who were exposed to high levels of modelled ambient air pollution reported high annoyance scores. Similarly, while Forsberg *et al*. reported high correlations between annoyance and NO_2_, they found no significant relationship between annoyance scores and SO_2_ concentrations in their study context [13]. When compared to our findings, the difference could be due to contextual factors. The Swedish study included several cities and towns across Sweden while this study is located in a relatively small and heavily industrial region with numerous petrochemical facilities.

We found significant correlation coefficients between odour annoyance and modelled pollutants (see Table 3). However, SO_2_ concentration had higher coefficient (r = 0.67) compared to NO_2_ (r = 0.56) at the census tract level. At the highly annoyed threshold (Figure 6), the results also showed that SO_2_ cumulative exposure–response curve had wider variation compared to the NO_2_ curve (Figure 5). These could likely be because individuals in the Sarnia area are more sensitive to SO_2_ pollution which has a unique pungent odour compared to NO_2_ which does not smell much. In this study, annoyance scores captured the within area variability of air pollution levels which suggests that odour annoyance is a function of real exposure and can be used as a proxy for air quality [13,15]. Consequently, where resources are limited, the establishment of expensive monitoring networks might not always be necessary because area variability of air pollution can be estimated in questionnaire surveys, where the marginal costs are low. The study showed that individual and mean census tract annoyance scores do reflect gradients of air pollution levels in Sarnia. However, the correlation coefficients between annoyance score and air pollution levels were improved when census tract level data were used. This finding suggests that exposure estimates based on census tract level would mitigate non-differential exposure misclassification [15,44]. We should however note that annoyance score can not replace personal exposure measurements, because of the high between-person variability of annoyance rating, which could partly be explained by the determinants of annoyance.

With the exception of gender, this study found no significant relationship between sociodemographic variables and odour annoyance scores (Tables 5 and 6). These findings are contrary to studies which reported, for example, that age, does significantly predict annoyance score reporting [10,45]. Luginaah *et al.* reported that young people were more likely to perceive odours and become annoyed than individuals who were older [26]. Furthermore, Steinheider [46] reported that age exerts an effect on olfactory sensitivity which results into the elderly having decreased sensitivity to odours. Although the descriptive statistics suggested a trend whereby older people were less likely to be annoyed by air pollution, the lack of significance in the multivariate analysis suggests the population in the Sarnia area which is constantly exposed to chronic odour and pollution may have been desensitized to exposure.

When examined in univariate analysis (not reported), health variables including self-assessed health status, chronic conditions, emotional distress, cardinal, and general health symptoms showed significant influence on the high annoyance reporting. However, when covariates were introduced, only cardinal symptoms reporting and their interaction with exposure to occupational dust showed significant influence on odour annoyance score reporting. These results signify the importance of covariates in modifying the influence of these health variables on odour annoyance reporting. Further, studies should explore how single health outcomes (e.g., asthma) rather than using composite health variables (e.g., chronic conditions) impact odour annoyance reporting. While examining the influence of individual health outcomes on self-reported air pollution problems, Piro *et al.* [9] found that people with chronic diseases (e.g., asthma, chronic heart disease) were more likely to report high odour annoyance.

Explaining the causal linkages between odour annoyance, odour perception, and health outcomes could be problematic. While examining the community reappraisal of perceived health effects of a petroleum refinery in Oakville, Ontario, Luginaah *et al.* [26] highlighted four possible causal mechanisms due to odour annoyance. First, there is a direct linkage between petrochemical facilities emissions and general health status. In fact, the industries were found to be significant contributors to the spatial variability of NO_2_ and SO_2_ pollution concentrations in Sarnia [33]. When compared, residents exposed to SO_2_ were more likely to report higher annoyance scores than when they are exposed to NO_2_ concentrations. This is partly because SO_2_ and its reduced compounds (e.g., hydrogen sulphide) are known to produce pungent smells detectable at very low concentration levels and can directly result in ill-health such as nausea or headache [26]. In this study, we found significant relationships between modelled NO_2_ and SO_2_ pollution concentration, cardinal symptoms reporting, and odour annoyance scores suggesting that odour annoyance is influenced by pollution levels and other covariates. This finding is consistent with Luginaah *et al.*’s who found that strong odours may result in ill-health (e.g., nausea) reporting [26]. Second, the relationship between exposure and general health status may be mediated by odour perception. In such instances, respondents who negatively perceive odours are sensitized and are more likely to report ill-health and attribute them to the numerous industrial facilities in the region. Third, the direction of the relationship could be the reverse, such that respondents experiencing ill-health are sensitized to believe and be annoyed by odours from the industrial facilities. Finally, the relationship could be bidirectional, such that odour perception and self-assessed health reporting are mutually reinforcing each other.

Despite the fact that the models used in this study captured the significant proportion of the spatial variability of NO_2_ and SO_2_ in Sarnia [33], there are some limitations worth mentioning. First, there is the possibility that annoyance levels might be related to other pollutants that might be correlated to NO_2_ and SO_2_ concentrations but were not measured in this study. The use of a 6-digit postal code instead of personal monitoring to develop air pollution estimates might also be another limitation. Nevertheless, the findings make important contributions to the literature.

In general, this study provides strong support for the second mechanism, whereby the relationship between exposure to industrial pollutants including NO_2_ and SO_2_, and odour annoyance reporting is mediated by odour perception and health outcomes [26,30]. This mediating role is supported by the fact that when we controlled for perception of odours (including the belief that industrial odours impact health and odour have not improved in the last five years), for example, the odds of reporting high annoyance actually increased. This finding suggests that covariates (e.g., odour perception) are key modifiers of high annoyance reporting. Nevertheless, other odour annoyance mechanisms, outlined above, are also possible given that they are not necessarily mutually exclusive and that evidence from these results is not sufficient to reject them either. For example, we found that residents who reported two or more cardinal symptoms were more likely to report high odour annoyance. This finding is similar to Luginaah *et al.*’s [26] who report that adult cardinal symptoms are strong predictors of odour perception.

The exposure–response relationships indicate that residents of Sarnia are annoyed with pollution concentrations levels that are very low compared to the Ontario 24 hour allowable NO_2_ and SO_2_ concentration guidelines. Considering the relationship between odours mediated mechanisms and health effects, the results suggest the need to revisit the set guidelines for allowable exposure to pollution to better protect residents. This finding is consistent with Amundsen *et al.* [41] who found that people in Norway are annoyed to exposure levels that are commonly occurring in European cities even though they “satisfy” national and international guidelines for outdoor air pollution.

In conclusion, questionnaire-based odour annoyance score of ambient air pollution can be a useful proxy for assessing the within-area variability of air quality and measure of perceived ambient exposure and could be used for evaluating the implementation of environmental policies. In fact, as a subjective score of air quality, odour annoyance has been incorporated in the Swedish National Monitoring programs [10]. Odour annoyance can also be utilized as a complementary tool for determining exposure and concerns of residents in sentinel high exposure environments like Sarnia. There is need for policy makers to pay attention to residents’ complaints and concerns regarding pollution exposure for better policy implementation. Although subjective, annoyance scores from industrial odours do capture the intra-urban variability of ambient pollution. Since this study, to the best of our knowledge, is one of the first to validate the use of odour annoyance score in a Canadian industrial context, there is need to conduct longitudinal studies across different contexts and scales to further validate the use of annoyance scores as proxies for air pollution if there is the potential to adopt them at the national level.

## Figures and Tables

**Table 1.** Mean and standard deviation of annoyance scores for males and females.

		Modelled Pollution			
		Low	Moderate	High	Total
		Mean Annoyance (SD) 1			
NO_2_	Male	1.42 (2.17)	2.92 (3.13)	2.97 (3.33)	2.57 (3.05)
	Female	3.38 (3.38)	3.51 (3.41)	4.69 (3.47)	3.75 (3.45)
	Total	2.53 (3.07)	3.25 (3.30)	3.86 (3.50)	3.22 (3.32)
	*t*-test value 2	4.583**	1.758*	2.576**	4.987**
SO_2_	Male	1.43 (2.22)	2.94 (3.19)	2.96 (3.20)	2.57 (3.05)
	Female	2.81(3.09)	3.92 (3.49)	4.38 (3.54)	3.75 (3.45)
	Total	2.19 (2.82)	3.47 (3.39)	3.76 (3.46)	3.22 (3.32)
	*t*-test value	3.505**	2.868**	2.859**	4.987**

^1^ SD = standard deviation;

^2^ The t-test was used to compare the male and the female mean annoyance scores.

* Significant at the 10% level;

** Significant at the 5% level.

**Table 2.** Mean and standard deviation of annoyance scores for different age groups.

		Modelled Pollution			
		Low	Moderate	High	Total
		Mean Annoyance (SD)			
NO_2_	18 – 24	2.83 (3.60)	2.89 (3.11)	4.64 (3.73)	3.40 (3.38)
	25 – 44	3.14 (3.45)	3.70 (3.27)	4.17 (3.82)	3.70 (3.43)
	45 – 64	2.69 (3.04)	3.59 (3.24)	4.07 (3.48)	3.44 (3.27)
	≥ 65	1.88 (2.76)	2.21 (3.17)	2.76 (2.85)	2.26 (2.99)
	Total	2.57 (3.07)	3.22 (3.27)	3.79 (3.48)	3.20 (3.30)
SO_2_	18 – 24	2.67 (4.62)	3.44 (3.32)	3.46 (3.55)	3.40 (3.38)
	25 – 44	2.65 (2.82)	4.02 (3.53)	3.94 (3.58)	3.70 (3.43)
	45 – 64	2.53 (2.97)	3.65 (3.30)	4.13 (3.38)	3.44 (3.27)
	≥ 65	1.47 (2.44)	2.40 (3.06)	2.85 (3.27)	2.26 (2.99)
	Total	2.26 (2.84)	3.44 (3.36)	3.67 (3.44)	3.20 (3.30)

**Table 3.** Pearson correlations between annoyance scores and individual and mean census tract level modelled pollution concentrations.

	Level of analysis	
Modelled pollutant	Individual (N = 774)	Census tract (N = 20)^1^
NO_2_	0.152**	0.563**
SO_2_	0.130**	0.666**

** Significant at the 5% level;

^1^ There were only 20 valid census tracts in which the census tract means were calculated.

**Table 4.** Univariate regression analysis between annoyance score and modelled ambient pollutants at the individual and census tract levels.

**Independent variable**	**Beta**	**Standard Error**	***t-*Value**	**Confidence interval**	***R*^2^**
**Individual Level**					
NO_2_	0.307	0.072	4.264**	0.166 – 0.449	0.023
SO_2_	0.280	0.077	3.656*	0.130 – 0.430	0.017
**Census tract level**					
NO_2_	0.378	0.131	2.892*	0.103 – 0.653	0.317
SO_2_	0.524	0.138	3.787**	0.233 – 0.814	0.443

* p-value < 0.001;

** p-value < 0.0001.

**Table 5.** Logistic regression results showing the relationship between annoyance and modelled NO_2_.

		**Logistic regression analysis Dependent variable: annoyance (high annoyance)**				
**Variables (reference)**	**Classification**	**Model I: OR (95% CI)**	**Model II: OR (95% CI)**	**Model III: OR (95% CI)**	**Model IV: OR (95% CI)**	**Model V: OR (95% CI)**
Nitrogen dioxide (1^st^ quartile, low)	2^nd^ Quartile	0.96 (0.51–1.81)	0.87 (0.45–1.68)	0.86 (0.45–1.67)	1.46 (0.64–3.28)	1.43 (0.63–3.24)
	3^rd^ quartile	1.89 (1.07– 3.34)*	1.84 (1.02–3.32)*	1.85 (1.02–3.36)*	3.06 (1.46–6.41)**	3.05 (1.45–6.40)**
	4^th^ quartile (high)	2.30 (1.32– 4.02)**	2.29 (1.28–4.11)**	2.31 (1.28–4.16)**	3.52 (1.68–7.39)**	3.32 (1.57–7.01)**
Age (18–24)	25–44		1.02 (0.45–2.30)	1.00 (0.44–2.29)	1.00 (0.38–2.61)	0.99 (0.38–2.63)
	45 – 64		0.77 (0.34–1.73)	0.80 (0.35– 1.81)	0.97 (0.37–2.56)	1.03 (0.38–2.74)
	65+		0.48 (0.20–1.16)	0.56 (0.23– 1.37)	0.77 (0.25–2.31)	0.81 (0.26–2.46)
Gender (Male)	Female		2.17 (1.41–3.33)***	2.35 (1.51–3.63)***	1.96 (1.17–3.27)*	1.84 (1.09–3.09)*
Exposure to dust at work (not exposed)	Exposed			1.57 (1.01–2.42)*	1.39 (0.82–2.34)	1.22 (0.70–2.13)
Employment (In the work force)	Not in work force				1.22 (0.70–2.13)	1.16 (0.66–2.04)
Health status (Very good/good/excellent)	Fair/poor				1.49 (0.81–2.72)	1.45 (0.78–2.69)
Cardinal symptoms (0–2 symptoms)	(≥3 symptoms)				1.84 (1.11–3.06)*	1.69 (0.98–2.92)
Odours affect health(Neutral/disbelieve)	Believe				4.96 (2.18–11.30)***	5.08 (2.22–11.62)***
Odours in last 5years (Improved)	Did not improve				1.75 (1.05–2.93)*	1.73 (1.03–2.90)*
Community satisfaction(satisfied)	dissatisfied				1.58 (0.96–2.61)	1.65 (0.99–2.75)
Coping with daily demands (able to cope)	Not able to cope				2.11 (1.07–4.16)*	2.06 (1.03–4.10)*
Exposure to dust x cardinal symptoms						4.87 (1.68–14.09)**
Goodness of fit 1		0.02	0.05	0.06	0.19	0.21
Cox & Snell R Square		0.02	0.04	0.05	0.15	0.16
Nagelkerke R Square		0.03	0.08	0.08	0.26	0.28

* p-value < 0.05;

** p-value < 0.01;

*** p-value < 0.001;

^1^ The goodness of fit is defined as one minus the ratio of the maximum log likelihood values of the fitted and constant only-term (null) models [43].

**Table 6.** Logistic regression results showing the relationship between annoyance and modelled SO_2_.

		**Logistic regression analysis Dependent variable: annoyance (high annoyance)**				
**Variables (reference)**	**Classification**	**Model I: OR (95% CI)**	**Model II: OR (95% CI)**	**Model III: OR (95% CI)**	**Model IV: OR (95% CI)**	**Model V: OR (95% CI)**
Sulphur dioxide (1^st^ quartile, low)	2^nd^ Quartile	2.08 (1.03–4.21)*	2.04 (1.00–4.15)*	2.01 (0.98–4.11)	1.87 (0.80–4.39)	1.72 (0.73–4.05)
	3^rd^ quartile	4.15 (2.16–7.98)***	3.67 (1.88–7.17)***	3.69 (1.89–7.23)***	4.01 (1.82–8.81)**	3.83 (1.73–8.47)**
	4^th^ quartile (high)	3.60 (1.85–6.98)***	3.30 (1.67–6.51)**	3.35 (1.70–6.63)***	4.18 (1.88–9.28)***	3.92 (1.76–8.74)**
Age (18–24)	25–44		1.28 (0.55–3.00)	1.24 (0.53–2.91)	1.13 (0.42–3.02)	1.17 (0.43–3.18)
	45–64		1.00 (0.43–2.31)	1.01 (0.44–2.36)	0.98 (0.37–2.61)	1.04 (0.39–2.81)
	65+		0.59 (0.2–1.47)	0.67 (0.27–1.67)	0.71 (0.24–2.14)	0.75 (0.24–2.28)
Gender (Male)	Female		2.06 (1.34–3.17)**	2.21 (1.43–3.43)***	1.80 (1.08–3.00)*	1.71 (1.02–2.86)*
Exposure to dust at work (not exposed)	Exposed			1.57 (1.02–2.44)*	1.31 (0.77–2.20)	1.12 (0.64–1.98)
Employment (In the work force)	Not in work force				1.24 (0.71–2.15)	1.19 (0.68–2.08)
Health status (Very good/good/excellent)	Fair/poor				1.65 (0.91–2.99)	1.63 (0.89–2.98)
Cardinal symptoms (0–2 symptoms)	(≥ 3 symptoms)				2.32 (1.39–3.88)**	2.17 (1.25–3.75)**
Odours affect health (Neutral/disbelieve)	Believe				5.33 (2.22–12.77)***	5.55 (2.31–13.35)***
Odours in last 5years(Improved)	Did not improve				1.80 (1.07–3.00)*	1.76 (1.05–2.96)*
Coping with daily demands(able to cope	Not able to				1.82 (0.92–3.62)	1.75 (0.87–3.49)
Exposure to dust x cardinal symptoms						4.79 (1.62–14.13)**
Goodness of fit 1		0.04	0.06	0.07	0.20	0.21
Cox & Snell R Square		0.03	0.05	0.06	0.16	0.17
Nagelkerke R Square		0.06	0.09	0.10	0.27	0.29

* p-value< 0.05;

** p-value< 0.01;

*** p-value< 0.001;

^1^ The goodness of fit is defined as one minus the ratio of the maximum log likelihood values of the fitted and constant only-term (null) models [43].

**Table 7.** Parameter estimate for NO_2_ and SO_2_ using ordinal logit model.

Parameter Estimates	Estimates					
	NO_2_ (ppb)	95% Confidence Interval		SO_2_ (ppb)	95% Confidence Interval	
		Lower	Upper		Lower	Upper
Threshold						
Little Annoyance	2.043	0.876	3.209	0.242	0.042	0.443
Highly Annoyance	4.399	3.198	5.600	2.069	1.812	2.327
Location						
Modelled Pollution	0.196	0.112	0.281	0.099	0.044	0.154

All results are statistically significant (p-value < 0.05); N = 774.
